# Antimicrobial activity of *Bauhinia tomentosa* and *Bauhinia vahlii* roots

**DOI:** 10.4103/0973-1296.66937

**Published:** 2010

**Authors:** Swarnalatha Dugasani, Madhu Katyayani Balijepalli, Satyanarayana Tandra, Mallikarjuna Rao Pichika

**Affiliations:** Department of Pharmacognosy and Phytochemistry, College of Pharmaceutical Sciences, Andhra University, Visakhapatnam - 530 003, India; 1 Department of Pharmaceutical Chemistry, International Medical University, Kuala Lumpur, 57000, Malaysia

**Keywords:** Antibacterial, antifungal, *Bauhinia tomentosa*, *Bauhinia vahlii*, minimal inhibition concentration, minimal microbicidal concentration

## Abstract

The hexane, ethylacetate and methanol extracts from *Bauhinia tomentosa* and *Bauhinia vahlii* roots were tested for their antimicrobial activity against Gram-positive bacteria (four strains), Gram-negative bacteria (three strains) and three fungi strains using microdilution methods, for the determination of minimal inhibition concentration (MIC) and the minimal microbicidal concentration (MMC). The MIC values of hexane extracts of *B. tomentosa* and *B. vahlii* roots were more than 250 µg/ml. The MIC values of ethylacetate and methanol extracts of *B. tomentosa* roots varied from 7.81 to 31.25 µg/ml and 31.25 to 62.50 µg/ml, respectively. The MIC values of ethylacetate and methanol extracts of *B. vahlii* roots varied from 15.63 to 62.5 µg/ml and 62.5 to 250 µg/ml, respectively. MMC values obtained are two times greater than the corresponding MIC values. The activities of ethylacetate extracts are attributed to the presence of flavonoids and that of methanol extracts are attributed to the presence of tannins.

## INTRODUCTION

The relatively large *Bauhinia* genus (family: Fabaceae) consisting of trees, climbers and shrubs is distributed in a wide range of geographic locations. Certain *Bauhinia* species have a long history of traditional medicinal applications.[1] *Bauhinia tomentosa* is a scrambling, many-stemmed shrub or small tree, the branches often dropping, with many slender twigs.[2] It is called as adavimandaramu in Telugu and phalgu in Sanskrit. It has been reported to contain amino acids,[3] proteins,[3] fatty acids,[4] minerals,[5] lectins,[6] protocatechuic acid,[7] phytohemagglutinins,[8] rutin,[9] quercetin[9] and isoquercetin.[10] *Bauhinia vahlii* is the largest creeper in India and is called adattige in Telugu and asamantaka in sanskrit. It has been reported to contain amino acids,[11] proteins,[11] minerals[12] and flavonoids.[13] Despite the very encouraging traditional medicinal applications of some species of *Bauhinia*, prior investigations to validate the traditional medicinal applications have not appeared in literature. The aim of this investigation was to evaluate the antimicrobial activity of the crude extracts of *B. tomentosa* and *B. vahlii* roots.

## MATERIALS AND METHODS

### Plant materials

The roots of *B. tomentosa* and *B. vahlii* were collected in July 2007 in Thalakona and Tirumala, respectively. Botanical identification of the plants was done by Dr. K. Madhava Chetty, Department of Botany, S.V. University, Tirupati. Specimens of *B. tomentosa* (Voucher number: 1821) and *B. vahlii* (Voucher number:1716) were conserved in S.V. University herbarium.

### Extraction of plant materials

The air-dried and powdered roots of *B. tomentosa* and *B. vahlii* (2 kg each) were extracted in Soxhlet apparatus sequentially with hexane, ethylacetate and methanol. The extracts were concentrated in a rotary evaporator under vacuum at a temperature not more than 50°C.

### Microbial strains

Three Gram-negative bacteria, four Gram-positive bacteria and two fungi obtained from American Type Culture Collection (ATCC) were used in this study. Three Gram-negative bacteria were *Escherichia coli* (ATCC 25922), *Pseudomonas aeruginosa* (ATCC 27853) and *Proteus vulgaris* (ATCC 12454); four Gram-positive bacteria were *Bacillus subtilis* (ATCC 10774), *Bacillus pumilus* (ATCC 14884), *Eneterococcus faecalis* (ATCC 2912) and *Staphylococcus aureus* (ATCC 25923) and the two fungi were *Candida krusei* (ATCC 6258) and *Candida albicans* (ATCC 10231).

### Culture media

Nutrient agar (NA) containing bromocresol purple was used for culturing of *Bacillus* species while NA was used for other bacteria. Sabouraud glucose agar was used for culturing fungi. Nutrient broth containing 0.05% phenol red and supplemented with 10% glucose (NBGP) was used for the determination of minimal inhibition concentration (MIC) and the minimal microbicidal concentration (MMC). The Muller Hinton agar (MHA) was also used for the determination of MMC.

### Reference antibiotics

Chloramphenicol (Sigma-Aldrich, USA) and Nystatin (Sigma-Aldrich) were used as reference antibiotics (RA) against bacteria and yeast, respectively.

### Minimal inhibition concentration and minimal microbicidal concentration determinations

The MIC of test samples and RA were determined as follows. The test sample was dissolved in dimethylsulfoxide (DMSO). The test and RA solutions obtained were added to NBGP and made to a final concentration 250 µg/ml. These solutions were serially diluted twofold to obtain concentration ranges of 0.49–250 µg/ml. Inoculums were standardized at 1.5 × 10^6^ CFU/ml by adjusting the optical density to 0.1 at 600 nm in a spectrophotometer. Each concentration was added in a well of 96-well microplate containing 95 l of NBGP and 5 µl of inoculum.[14] The final concentration of DMSO in the well was less than 1%. Preliminary analysis with 1% (v/v) DMSO/NBGP did not affect either the growth of the test organisms or the change in color due to microbial growth. The negative control well consisted of 195 µl of NBGP and 5 µl of the standard inoculum.[14] The plates were covered with a sterile plate sealer, then agitated to mix the content of the wells using a plate shaker and incubated at 37°C for 24 h. The assay was repeated thrice. Microbial growth was determined by observing the change of color in the wells. The color was red where there was no growth and yellow when there was growth. The lowest concentration showing no color change was considered as MIC.

For the determination of MMC, 5 µl of liquid from each well that showed no change of color was plated on MHA and incubated at 37°C for 24 h. The lowest concentration that yielded no growth after this subculturing was taken as MMC.

## RESULTS AND DISCUSSION

The antibacterial and antifungal activities of the crude extracts were evaluated and the results are reported in Tables 1 and 2. The MIC values of hexane extracts from *B. tomentosa* and *B. vahlii* roots on all microbial strains were greater than 250 µg/ml. The MIC values of ethylacetate and methanol extracts of *B. tomentosa* and *B. vahlii* roots were less than 250 µg/ml. For ethylacetate extract of *B. tomentosa* roots, MIC values on Pr. vulgaris, S. aureus and other strains were 7.81, 31.25 and 15.63, µg/ml respectively. For ethylacetate extract of *B. vahlii* roots, MIC values on Pr. vulgaris, S. aureus and other strains were 15.63, 62.5 and 31.25 µg/ml, respectively. For methanol extract of *B. tomentosa* roots, the MIC values on S. aureus and other strains were 62.50 and 31.25 µg/ml, respectively. For methanol extract of *B. vahlii* roots, MIC values on Pr. vulgaris, S. aureus and other strains were 62.5, 250 and 125 µg/ml, respectively. The results of MIC [Table 1] and MMC [Table 2] showed that the MMC values obtained are two times higher than MIC on the corresponding sensitive microorganisms.

**Table 1:** MICs (g/ml) of the hexane, ethylacetate and methanol extracts from *B. tomentosa* and *B. vahlii* roots and reference antibiotics

Microogranisms	*B. tomentosa* extracts			*B. vahlii* extracts			RAª
	Hexane	Ethylacetate	Methanol	Hexane	Ethylacetate	Methanol
Gram-negative bacteria							
* Es. coli*	>250	15.63	31.25	>250	31.25	125	15.63
* Ps. aeruginosa*	>250	15.63	31.25	>250	31.25	125	3.90
* Pr. vulgaris*	>250	7.81	31.25	>250	15.63	62.5	7.81
Gram-positive bacteria							
* B. subtilis*	>250	15.63	31.25	>250	31.25	125	7.81
* B. pumilus*	>250	15.63	31.25	>250	31.25	125	7.81
* En. faecalis*	>250	15.63	31.25	>250	31.25	125	7.81
* S. aureus*	>250	31.25	62.50	>250	62.5	250	15.63
Fungi							
* C. krusei*	>250	15.63	31.25	>250	31.25	125	7.81
* C. albicans*	>250	15.63	31.25	>250	31.25	125	7.81

ª Reference antibiotics (chloramphenicol for bacteria; nystatin for fungi)

**Table 2:** MICs (g/ml) of the hexane, ethylacetate and methanol extracts from *B. tomentosa* and *B. vahlii* roots and reference antibiotics

Microogranisms	*B. tomentosa* extracts			*B. vahlii* extracts			RAª
	Hexane	Ethylacetate	Methanol	Hexane	Ethylacetate	Methanol
Gram-negative bacteria							
* Es. coli*	nd	31.25	62.5	nd	62.5	250	7.81
* Ps. aeruginosa*	nd	31.25	62.5	nd	62.5	62.5	1.95
* Pr. vulgaris*	nd	15.63	15.63	nd	31.25	125	3.90
Gram-positive bacteria							
* B. subtilis*	nd	31.25	62.5	nd	62.5	250	3.90
* B. pumilus*	nd	31.25	62.5	nd	62.5	250	3.90
* En. faecalis*	nd	31.25	62.5	nd	62.5	250	3.90
* S. aureus*	nd	62.5	125	nd	125	> 250	7.81
Fungi							
* C. krusei*	nd	31.25	62.5	nd	62.5	250	3.90
* C. albicans*	nd	31.25	62.5	nd	62.5	250	3.90

ª Reference antibiotics (chloramphenicol for bacteria; nystatin for fungi); nd: not determined because MIC > 250 µg/ml

In conclusion, it can be said that the extracts of *B. tomentosa* roots were more potent than the respective extracts of *B. vahlii* roots. The relative potencies of extracts are in the order of ethylacetate > methanol > hexane.

Preliminary chemical examination showed the presence of sterols and terpenoids in hexane extracts, flavonoids in ethylacetate extracts, glycosides and tannins in methanol extracts of *B. tomentosa* and *B. vahlii* roots.

Only the antimicrobial activity of *B. tomentosa* leaves has been reported in the literature.[15] To the best of our knowledge, antimicrobial activitiy of *B. tomentosa* and *B. vahlii* roots is being reported for the first time. Nevertheless, this study supports the traditional use of *B. tomentosa* roots as a vermifuge[16,17] and as a mouth gargle.[18] In addition, a good number of research papers documented the antimicrobial potency of some species of *Bauhinia*^1^ and the activity is attributed to the presence of flavonoids[19] and phenolic compounds.[20–21]

Considering the medicinal importance of the tested microorganisms, the results of this study are considered to be very promising in the perspective of new drug discovery from plant sources. *Ps. aeruginosa* has emerged as one of the most problematic Gram-negative pathogens, with the alarmingly high antibiotic resistance rates.[22] Even with the most effective antibiotics against this pathogen, the resistance rates were detected as 15–20.4% amongst 152 *Ps. aeruginosa* strains. This pathogen was found to be sensitive to the crude extracts of *B. tomentosa* and *B. vahlii* roots. *Bacillus* species and *Es. coli* are agents of food poisoning.[23,24]

*C. albicans* and other *Candida* species, causing candidiasis, are increasingly important as they are distributed worldwide, and also they are frequent opportunistic pathogens in AIDS patients.[25] *Proteus* species are widespread in environment and account for 3% of nosocomial infections in the United States.[26] The incidence of enterococcal bacteremia due to *En. faecalis* is continuously increasing.[27] *S. aureus* has been associated with persistent and antibiotic-resistant infections.[28]

Our investigation showed that the antimicrobial activity of ethylacetate extracts from *B. tomentosa* and *B. vahlii* roots might be related to the presence of flavonoids. Flavonoids are known to exhibit antimicrobial activity through formation of a complex with the bacterial cell wall. The antimicrobial activity of the methanol extracts from *B. tomentosa* and *B. vahlii* roots might be attributed to the presence of tannins. The probable mechanism of these phenolic compounds activity includes enzyme inhibition by oxidized compounds, possibly through reaction with sulfhydryl groups or through more nonspecific interactions with proteins.[29]

Finally, antimicrobial activity of the extracts from *B. tomentosa* and *B. vahlii* roots may be due to the presence of both antifungal and antibacterial compounds in them. The present study provides an important basis for the use of extracts from these plants for the treatment of infections associated with the studied microorganisms. Isolation and characterization of bioactive compounds from these two species of genus *Bauhinia* is currently being carried out in our laboratory.
